# A new cytoplasmic interaction between junctin and ryanodine receptor Ca^2+^ release channels

**DOI:** 10.1242/jcs.160689

**Published:** 2015-03-01

**Authors:** Linwei Li, Shamaruh Mirza, Spencer J. Richardson, Esther M. Gallant, Chris Thekkedam, Suzy M. Pace, Francesco Zorzato, Dan Liu, Nicole A. Beard, Angela F. Dulhunty

**Affiliations:** John Curtin School of Medical Research, ACT 0200, Australia

**Keywords:** Junctin, ASPH, Ryanodine receptor, RyR, Sarcoplasmic reticulum, Cytoplasmic interaction, Luminal interaction

## Abstract

Junctin, a non-catalytic splice variant encoded by the aspartate-β-hydroxylase (*Asph*) gene, is inserted into the membrane of the sarcoplasmic reticulum (SR) Ca^2+^ store where it modifies Ca^2+^ signalling in the heart and skeletal muscle through its regulation of ryanodine receptor (RyR) Ca^2+^ release channels. Junctin is required for normal muscle function as its knockout leads to abnormal Ca^2+^ signalling, muscle dysfunction and cardiac arrhythmia. However, the nature of the molecular interaction between junctin and RyRs is largely unknown and was assumed to occur only in the SR lumen. We find that there is substantial binding of RyRs to full junctin, and the junctin luminal and, unexpectedly, cytoplasmic domains. Binding of these different junctin domains had distinct effects on RyR1 and RyR2 activity: full junctin in the luminal solution increased RyR channel activity by ∼threefold, the C-terminal luminal interaction inhibited RyR channel activity by ∼50%, and the N-terminal cytoplasmic binding produced an ∼fivefold increase in RyR activity. The cytoplasmic interaction between junctin and RyR is required for luminal binding to replicate the influence of full junctin on RyR1 and RyR2 activity. The C-terminal domain of junctin binds to residues including the S1–S2 linker of RyR1 and N-terminal domain of junctin binds between RyR1 residues 1078 and 2156.

## INTRODUCTION

Contraction in the heart and skeletal muscle depends on the release of Ca^2+^ from the intracellular sarcoplasmic reticulum (SR) Ca^2+^ store. Inherited or acquired changes in Ca^2+^ release lead to skeletal and cardio-myopathies and cardiac death (Györke and Carnes, 2008). The efficacy of Ca^2+^ release depends an influence of the ‘Ca^2+^ load’ in the SR on the activity of ryanodine receptor (RyR) Ca^2+^ release channels, which is facilitated by a Ca^2+^-dependent interaction between CSQ proteins (also known as CASQ) and the RyR through the ‘anchoring’ protein junctin, which is a non-catalytic splice variant encoded by the aspartate-β-hydroxylase (*Asph*) gene (Dulhunty et al., 2009; Dulhunty et al., 2012; Wei et al., 2009a). A second ‘anchoring’ protein, triadin also binds to CSQ and the RyR, but does not transmit signals from CSQ to the RyR in skeletal muscle *in vitro* (Wei et al., 2009a), although it does influence Ca^2+^ release during excitation–contraction coupling in intact myotubes (Goonasekera et al., 2007) and anchoring of CSQ to the junctional SR membrane (Boncompagni et al., 2012). Different isoforms of the RyR, CSQ and triadin are expressed in cardiac and skeletal muscle, whereas the same isoform of junctin is expressed in both muscle types.

**Fig. 1.**
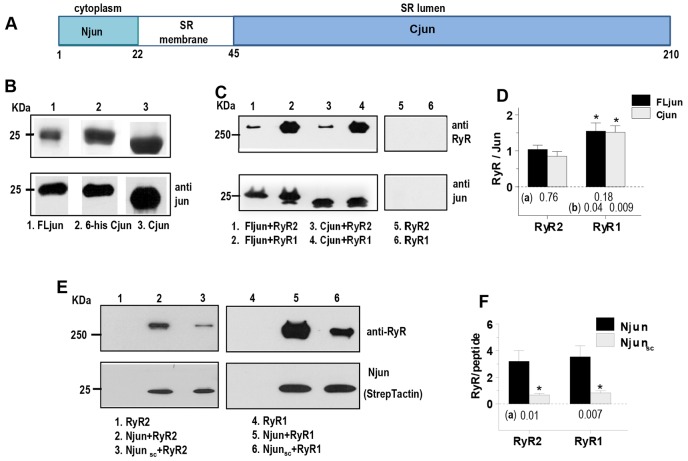
**Junctin structure and binding to RyRs.** (A) Junctin topology showing the cytoplasmic N-terminal domain (Njun), the transmembrane and C-terminal domains (Cjun). (B) Purification of FLjun and recombinant Cjun. The upper panel shows a Coomassie-stained SDS-PAGE gel with FLjun (lane 1), His_6_ (6-his)-tagged Cjun (lane 2) and Cjun after His tag removal (lane 3). The lower panel shows a western blot (anti-junctin antibody). (C) Co-immunoprecipitation of RyR1 by FLjun and 6-his Cjun, or RyR2 by FLjun and by 6-his Cjun (lanes 1 to 4). No RyR1 or RyR2 bound to the beads or the anti-junctin antibody in the absence of FLjun or Cjun (lanes 5 and 6). (D) Mean±s.e.m. densitometry values of RyR:FLjun or RyR:Cjun (RyR2, *n* = 5; RyR1, *n* = 4). *P<0.05 for RyR2 values significantly less than RyR1; *P*-values below bins are for differences between (a) FLjun and Cjun bound to RyR2 or RyR1, and (b) FLjun bound to RyR1 and FLjun bound to RyR2, or Cjun bound to RyR1 and Cjun bound to RyR2. (E) Co-immunoprecipitation of RyR2 and RyR1 by biotinylated Njun or biotinylated scambled Njun peptide (Njun_sc_). (F) Mean±s.e.m. densitometry values for RyR:Njun or RyR:Njun_sc_ (RyR2, *n* = 7; RyR1, *n* = 8). *P<0.05 for RyR:Njun_sc_ values significantly less than RyR:Njun; *P*-values below bins (a) are for differences between Njun and Njun_sc_ binding to RyR2 or RyR1.

The functions of junctin, triadin and CSQ in SR Ca^2+^ signalling have been extensively explored in transgenic animals. Triadin-, junctin- or CSQ-null animals survive, but their longevity and ability to tolerate stress is compromised (Altschafl et al., 2011; Dainese et al., 2009; Oddoux et al., 2009). Thus, normal expression of each of the proteins is required for normal function. In contrast to the transgenic studies there are few reports addressing the molecular interactions between either triadin or junctin and the RyR *in vitro*. The regions of junctin that support functional interactions with RyRs are unknown. Junctin contains a short N-terminal cytoplasmic domain, a single SR membrane-spanning domain and a longer luminal C-terminal domain (Fig. 1A) that contains binding sites for CSQ and the RyR (Altschafl et al., 2011; Kobayashi et al., 2000). The binding site for junctin on RyR1 has not been determined, but two junctin-binding regions on luminal domains of RyR2 have been identified (Altschafl et al., 2011). Our aim here has been to examine the functional interactions between junctin and skeletal RyR1 and cardiac RyR2 channels and to explore the role of the cytoplasmic and luminal domains of junctin in modulating RyR activity. The results show the expected interaction between the luminal domain of junctin (as well as one of its KEKE motifs) with the luminal, but not the cytoplasmic domains, of RyR1 and RyR2 channels. In addition we find an unexpected and substantial interaction between the cytoplasmic domain of junctin and the cytoplasmic, but not the luminal, domains of RyR1 and RyR2. The cytoplasmic interaction appears to be essential to reproduce the effect of full-length junctin on the RyR channels.

## RESULTS

### Full-length junctin, and its C-terminal and N-terminal domains bind to RyR1 and RyR2

Co-immunoprecipitation and affinity chromatography were used to determine the molecular nature of the association between junctin and RyRs. RyR2 purified from sheep heart or RyR1 purified from rabbit skeletal muscle were coupled to anti-RyR1-antibody-conjugated protein-A/G–agarose and full-length junctin (FLjun; isolated from skeletal muscle) or C-teminal domain of canine junctin (Cjun, expressed in *E. coli*) were coupled to anti-junctin-antibody-conjugated protein-A/G–agarose, to assess binding to RyRs. FLjun bound to both RyR2 and RyR1 as assessed by anti-junctin co-immunoprecipitation (Fig. 1C, lanes 1 and 2), or when RyRs were conjugated to anti-RyR-antibody-conjugated protein-A/G–agarose (*n* = 2–3, included in averages in Fig. 1D). There was similar association between Cjun and RyR2 or RyR1, as assessed by both anti-RyR (Fig. 1C, lanes 3 and 4) and anti-junctin antibody co-immunoprecipitation (*n* = 2–3, included in averages in Fig. 1D). Comparable amounts of Cjun and FLjun bound to the RyRs (Fig. 1D). The density of RyR2 bands was lower than RyR1 owing to a reduced antibody affinity for RyR2. No FLjun, Cjun, RyR1 or RyR2 associated with protein-A/G–agarose in the absence of antibody, or in the presence of antibody but absence of epitope (e.g. Fig. 1C, lanes 5 and 6).

We next assessed the binding between RyRs and the cytoplasmic N-terminal domain of junctin (Njun), using co-immunopreciptation of RyR2 by biotinylated Njun (Fig. 1E). As it was assumed that the RyR interactions with junctin occurred only between the luminal domains of the proteins, it was surprising that RyR1 and RyR2 bound to the biotinylated Njun peptide (Fig. 1E, lanes 2 and 5). Given that RyRs isolated from heart or skeletal muscle bound to NeutrAvidin agarose beads, but recombinant RyR1 and RyR2 did not (Fig. 1E, lanes 1 and 4), recombinant channels were used in this experiment. However a similar interaction between muscle-isolated RyRs and the cytoplasmic Njun domain is supported by the functional and binding data described below. We did find that some RyR bound to a scrambled Njun peptide (Fig. 1E, lanes 3 and 6), but this was <20% of that bound by Njun (Fig. 1F). Neither adding 1% Triton X-100 to the wash buffer, nor increasing the volume of wash buffer or number of washes, altered the binding. This limited binding was considered to be either non-specific or non-functional as the scrambled peptide did not alter channel activity (see Fig. 3). It is perhaps not surprising that scrambled Njun (with an isoelectric point of 9.70, containing two glutamic acids, three lysines and three histadines) associated weakly and non-specifically with RyRs, without functional consequences, as the large cytoplasmic domain of the RyR contains hydrophilic surfaces. Attempts at complimentary co-immunoprecipitation of Njun by RyRs was unsuccessful as the Njun peptide bound to protein-A/G–agarose (*n* = 4).

### Full-length junctin activates RyR2 and RyR1 channels from heart and skeletal muscle

**Fig. 2.**
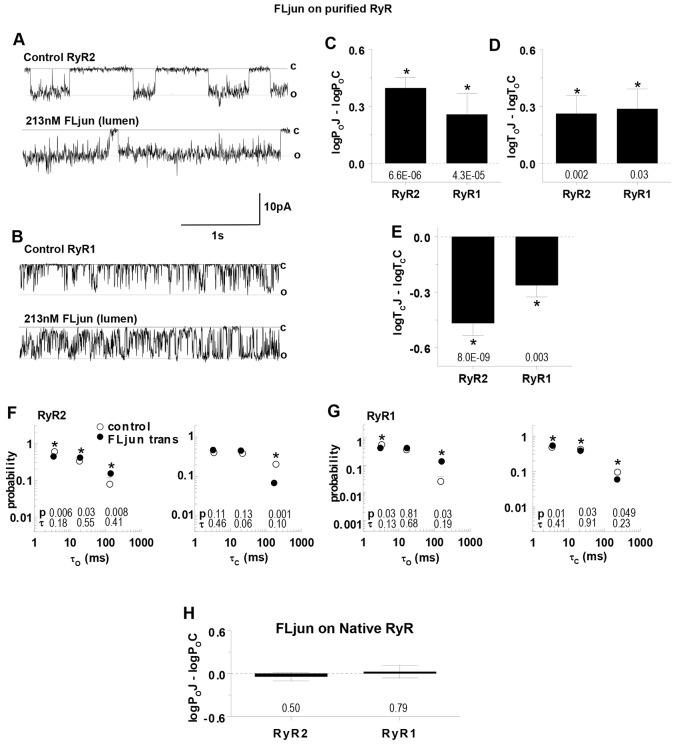
**Activation of purified RyR2 and RyR1 channels by luminal FLjun.** (A,B) 3 s of purified RyR activity at −40 mV. Channel opening is downward, from zero current (continuous line, c) to maximum single-channel conductance (broken line, o). The upper trace in each panel shows control activity and the lower trace after adding 213 nM (5 µg/ml) FLjun to the luminal solution bathing RyR2 (A) or RyR1 (B). (C–E) Mean±s.e.m of relative open probability (log*P*_o_J–log*P*_o_C), mean open time (log*T*_o_J–log*T*_o_C) and mean closed time (log*T*_c_J–log*T*_c_C), determined for individual RyR2 (*n* = 14) and RyR1 (*n* = 10) channels. (F,G) Effects of FLjun on average open (left) and closed (right) time constants and fraction of events in each time constant (Sigworth and Sine, 1987). The probability of events falling into each time constant is plotted against the time constant (in ms), for control data (open circles) and with FLjun (filled circles) for RyR2 (F) or RyR1 (G). Horizontal and vertical bars indicate the s.e.m. for the time constant and probability, respectively, and are contained within the symbols if not visible; *P*-values below bins test are for differences between the probability of events (*P*, upper row) or time constant (τ, lower row) with FLjun and control. *P<0.05 between control and FLjun data. (H) Mean±s.e.m. open probability of native RyR2 (*n* = 8) and RyR1 channels (*n* = 12) exposed to FLjun, relative to open probability prior to exposure; *P*-values below the bins in C–E and in H are for differences between FLjun and control parameters.

RyR channels (both native RyR channels in SR vesicles or RyR channels purified from SR vesicles) added to the solution on the cis side of the lipid bilayer incorporate into bilayers with their cytoplasmic side facing that solution and luminal side facing the trans solution (Beard et al., 2002; Laver, 2005). Therefore these solutions are referred to as cytoplasmic and luminal solutions, respectively. The average conductances for the RyR channels were 284.8±9.1 pS for RyR1 or 266.6±10.5 for RyR2, which is in the range reported previously with physiological [Ca^2+^]s of 1 µM cis and 1 mM trans (Laver et al., 1995; Tinker and Williams, 1993; Wei et al., 2009a). Channel activity increased when FLjun was added to the luminal solution at a maximal activating concentration of 213 nM [the molar equivalent of 5 µg/ml, as used previously (Wei et al., 2009a)] (Fig. 2A–C), associated with an increase in mean open time and a decrease in mean closed time (Fig. 2D,E). The open and closed time distributions (τ_o_ and τ_c,_ respectively) were described by three time constants (τ_1,_ from 1–10 ms; τ_2_, from 10–50 ms or τ_3_, from 50–500 ms) that were not altered by FLjun, although there was a significant increase in long openings in τ_o3_ (and fewer in τ_o1_), and a decrease in long closures in τ_c3_ (with more in τ_c1_) (Fig. 2F,G).

**Fig. 3.**
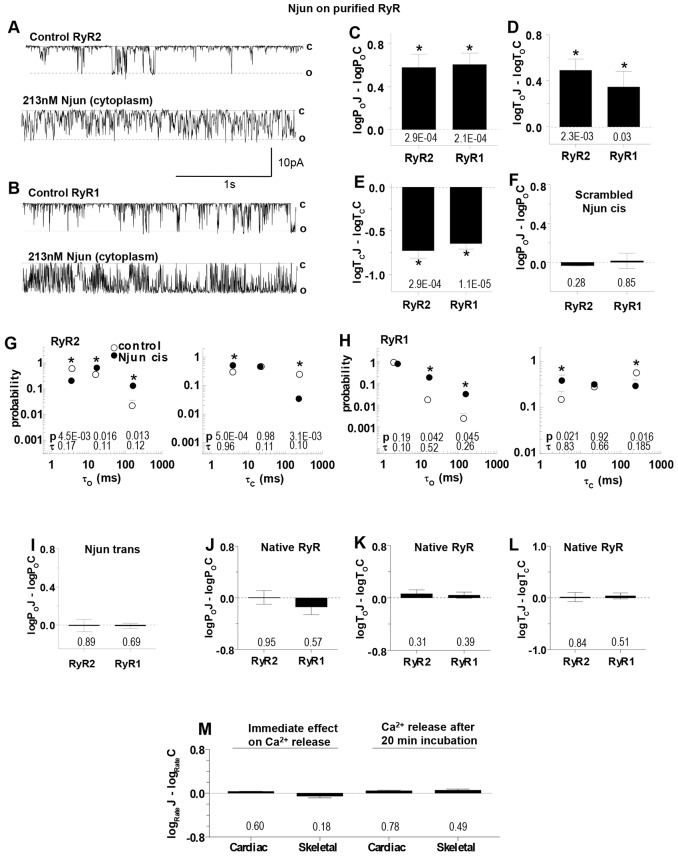
**Cytoplasmic Njun activates purified RyR2 and RyR1 channels.** (A,B) Records of 3 s of purified RyR channel activity at −40 mV as described in Fig. 2. The upper trace in each panel shows control activity and the lower trace activity in the same channel after adding cytoplasmic 213 nM Njun to RyR2 (A) and RyR1 (B). (C–E) Mean±s.e.m log*P*_o_J–log*P*_o_C, log*T*_o_J–log*T*_o_C and log*T*_c_J–log*T*_c_C, as defined in the legend to Fig. 2, for RyR2 (*n* = 14) and RyR1 (*n* = 10). (F) Mean±s.e.m. relative open probability after adding 213 nM scrambled Njun to the cytoplasmic solution for RyR2 (*n* = 8) or RyR1 (*n* = 16). (G,H) Effects of 213 nM Njun on average open (left) and closed (right) time constants. The probability of events falling into each time constant is shown for control (open circles) and with 213 nM Njun (filled circles) for RyR2 (G) or RyR1 (H). Further details, including definition of *P*-values above the *x*-axis are described in the legend to Fig. 2. (I) Mean±s.e.m. relative open probability after adding 213 nM Njun to the luminal solution for RyR2 (*n* = 8) or RyR1 (*n* = 14). (J–L) Mean±s.e.m. relative open probability after adding 213 nM Njun to the cytoplasmic side of native RyRs – values are log*P*_o_J–log*P*_o_C, log*T*_o_J–log*T*_o_C and log*T*_c_J–log*T*_c_C for RyR2 (*n* = 13) and RyR1 (*n* = 14). (M) The rate of Ca^2+^ release from Ca^2+^-loaded SR vesicles after blocking SERCA with thapsigargin. Left, immediate effect of Njun on cardiac SR (*n* = 4), and skeletal SR (*n* = 4). Right, effect of preincubation with 5 µM Njun on caffeine induced Ca^2+^ release through RyR2 (5 mM caffeine, *n* = 7) and RyR1 (0.5 mM caffeine, *n* = 11). *P<0.05 between control and junctin construct data. In C–F, I–L and M, *P*-values for differences between Njun or scrambled Njun and control are given below the bins.

**Fig. 4.**
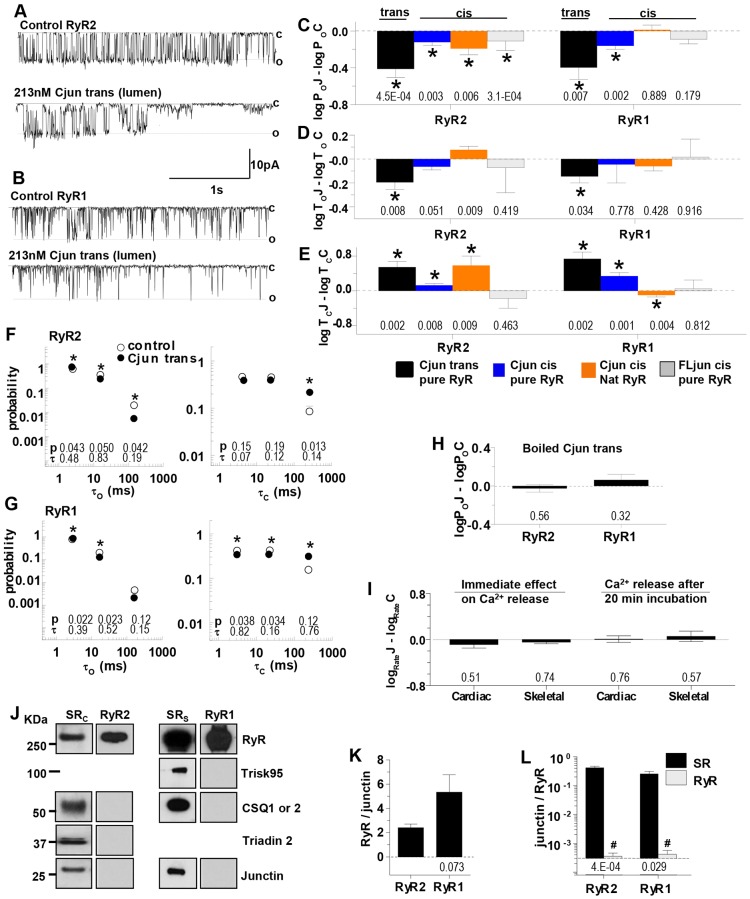
**Cjun inhibits native RyR2 and RyR1 channels.** (A,B) The upper trace in each panel shows control activity and the lower trace activity after adding 213 nM Cjun to the luminal solution for 3 s at −40 mV as described in Fig. 2. (C–E) Mean±s.e.m log*P*_o_J–log*P*_o_C, log*T*_o_J–log*T*_o_C and log*T*_c_J–log*T*_c_C, as defined in the legend to Fig. 2, for luminal addition of Cjun to purified RyR2 (*n* = 18) and RyR1 (*n* = 16) (black bins); cytoplasmic Cjun on purified RyR2 (*n* = 32) and RyR1 (*n* = 12) (blue bins); cytoplasmic Cjun on native RyR2 (*n* = 8) and RyR1 (*n* = 6) (orange bin); and cytoplasmic recombinant FLjun on purified RyR2 (*n* = 12) and RyR1 (*n* = 14) (grey bin). (F,G) Effects of luminal 213 nM Cjun on average open (left) and closed (right) time constants as described for Fig. 2. *P<0.05 for differences between the fraction of events in a particular time constant group under control conditions and with 213 nM Cjun. (H) Mean±s.e.m. of luminal 213 nM boiled Cjun on relative open probability of RyR2 (*n* = 5) and RyR1 (*n* = 10). (I) Effect of 5 µM Cjun on the relative rate of Ca^2+^ release from SR vesicles immediately after application (left, cardiac *n* = 5; skeletal *n* = 5) and on caffeine-induced Ca^2+^ release after 20 min pre-incubation and 10 min exposure during Ca^2+^ loading (right, cardiac *n* = 5; skeletal *n* = 5). *P*-values for differences between rates with Cjun and buffer are given below the bins. (J) Western blots probed for RyR, skeletal triadin (Trisk95), CSQ, triadin 2 and junctin. From left to right: cardiac SR (SR_C_), purified RyR2, skeletal SR (SR_sk_), and purified RyR1. (K,L) Mean±s.e.m. densitometry data from western blots of SR and purified RyR from cardiac (*n* = 4) and skeletal (*n* = 4) muscle. Mean±s.e.m. RyR:junctin; the *P*-value for the difference between cardiac and skeletal SR is give below the bin (K). Junctin:RyR ratios show no significant junctin density in the purified preparations; *P*-values for differences between SR and purified RyR are given below the bins; ^#^*P*<0.05, reflecting less junctin (L).

In control experiments, FLjun was added to the luminal side of native RyR channels that remained associated with endogenous junctin (see Fig. 4 below and Wei et al., 2009a). The rationale was that if endogenous junctin was bound to native RyRs, then exogenous FLjun added to the luminal solution could not bind to or activate the channels. Consistent with this hypothesis, there was no significant effect of luminal FLjun on either native RyR1 or native RyR2 channels (Fig. 2H).

### The N-terminal domain of junctin activates RyR channels

Njun added to the solution on the cytoplasmic side of the lipid bilayer dramatically increased RyR activity (Fig. 3A,B). RyR2 open probability increased 6.9±2.4-fold and RyR1 increased 5.0±1.0-fold (Fig. 3C). This was significantly greater than the 2.8±0.44 (RyR2, *P* = 1.2×10^−^^3^) and 1.9±0.14 (RyR1, *P* = 1.9×10^−4^) increases produced by luminal FLjun, but was similarly due to longer open times (Fig. 3D) and briefer closures (Fig. 3E). As with FLjun, there was no change in the open or closed time constant values, but a decrease in the number of longest closed events and increase in the number of longest opening events (Fig. 3G,H). Channel activity was not altered by addition of the scrambled cytoplasmic Njun peptide (Fig. 3F) or by luminal Njun (Fig. 3I), indicating that there is a specific action of Njun on the cytoplasmic side of the RyRs.

To assess whether Njun was bound to RyR1 *in vivo*, we again utilised native RyR channels in SR vesicles containing endogenous junctin. As with FLjun, the rationale was that if the N-terminal domain of endogenous junctin was bound to native RyRs, then exogenous Njun added to the cytoplasmic solution could not bind to the channels. Indeed there was no significant change in relative gating parameters after adding 213 nM Njun to the cytoplasmic side of native RyRs resident in the membrane of SR vesicles (Fig. 3J–L). We also examined the effects of Njun on Ca^2+^ release from SR vesicles, through native RyRs, which are oriented with their cytoplasmic surface facing the extravesicular solution (Saito et al., 1984). Njun was added at 5 µM because high peptide concentrations are required to instantly alter Ca^2+^ release from the SR (Dulhunty et al., 1999). Consistent with the single channel results, Njun did not immediately alter resting Ca^2+^ release from cardiac or skeletal SR (Fig. 3M, left). Similarly, 20 min pre-incubation in 5 µM Njun (plus 10 min exposure during Ca^2+^ loading steps) did not alter caffeine-induced Ca^2+^ release (Fig. 3M, right), which reflects the physiological Ca^2+^-induced Ca^2+^ release process (Pagala and Taylor, 1998). Therefore, we suggest that most Njun-binding sites are occupied by the N-terminal tail of endogenous junctin and thus that Njun binding to RyRs might occur *in vivo*.

### The C-terminal domain of junctin inhibits RyR channels

We expected that luminal Cjun would activate RyRs in the same way as FLjun, due to an assumed dominance of the luminal interaction between the proteins. Contrary to expectation Cjun inhibited the channels (Fig. 4A–C, solid black bins). The open probability (*P*_o_) fell to 0.48±0.06 of control in RyR2 and to 0.68±0.17 in RyR1, due to a small reduction in open times (Fig. 4D) and a larger increase in closed times (Fig. 4E). The open and closed time constants were not affected by Cjun, but there were fewer long open events and more long closed events (Fig. 4F,G). This was likely to be a specific effect of the native Cjun because denaturing the protein by boiling for 10 min removed its effects on channel open probability (Fig. 4H).

Cjun added to the cytoplasmic (cis) solution caused a small significant decline in channel activity (Fig. 4C–E, blue bins), which was significantly less than the fall in activity with Cjun in the luminal solution (RyR2, *P* = 1.8×10^−^^3^; RyR1, *P* = 0.047). This was not due to Cjun crossing the bilayer to access luminal RyR domains, as subsequent addition of luminal Cjun produced normal inhibition and, conversely, luminal addition of Cjun did not prevent the effect of subsequent cytoplasmic addition (*n* = 6, RyR1 and *n* = 12, RyR2). To determine whether the C-terminal domain of endogenous junctin bound to the cytoplasmic side of native RyRs, cytoplasmic Cjun was added to native channels where it inhibited RyR2 but not RyR1 (Fig. 4C–E, orange bins). In contrast, however, the rate of Ca^2+^ release from SR vesicles was unaffected by 5 µM Cjun either immediately after addition or during caffeine-induced Ca^2+^ release after a 20 min pre-incubation plus 10 min exposure during Ca^2+^ loading (Fig. 4I). Finally, recombinant FLjun with a His-tag-protected N-terminus added to the cytoplasmic side of purified channels, did not consistently change gating (Fig. 4C–E, grey bins). These inconsistent results suggest that any cytoplasmic effect of Cjun is weak and non-specific. As with scrambled Njun, this is not surprising given the overall highly charged nature of both Cjun and the cytoplasmic RyR domains.

The strong action of Cjun on the luminal side of purified RyRs was not due to the peptide binding to residual CSQ associated with the solubilised RyR as there is no detectable CSQ, triadin or junctin in these preparations (Fig. 4J–L).

### Combined actions of Cjun and Njun on RyR channels

**Fig. 5.**
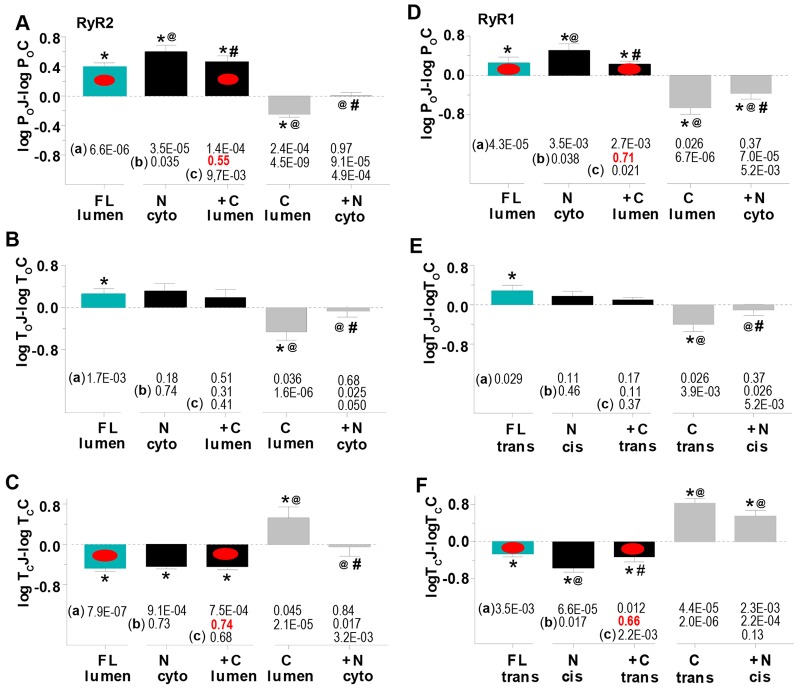
**Channel activity after adding Njun to the cytoplasmic solution, then Cjun to the luminal solutions, reproduces activity seen after adding FLjun to the luminal solution.** (A–F) Data for three different experiments is compared in each graph. First, 213 nM FLjun added alone to the luminal solution (FL lumen, cyan bins – data from Fig. 2, included for comparison). Second, addition of 213 nM Njun cytoplasmically, then 213 nM Cjun to the luminal solution (N cyto, +C lumen, respectively, black bins, RyR2, *n* = 10; RyR1, *n* = 10). Third, addition of 213 nM Cjun luminally, then 213 nM Njun to the cytoplasmic solution (C lumen, +N cyto, respectively, grey bins, RyR2, *n* = 8; RyR1, *n* = 10). (A,D) log*P*_o_J–log*P*_o_C; (B,E) log*T*_o_J–logT_o_C; (C,F) log*T*_c_J–logT_c_C, as defined in the legend to Fig. 2. *P<0.05 between control parameters and with 213 nM FLjun, Cjun or Njun; *P*-values are given beneath each row (a). ^@^*P*<0.05 significant difference between parameters with luminal FLjun and with cytoplasmic Njun or luminal Cjun, *P*-values are given beneath each row (b). ^#^*P*<0.05 for adding luminal Cjun after cytoplasmic Njun (2nd black bin), or adding cytoplasmic Njun after luminal Cjun (2nd grey bin), *P*-values are given beneath each row (c). The red dots indicate changes in open probability and mean closed time that are not significantly different (*P*-values in red font) with luminal addition of FLjun alone or with luminal Cjun added after cytoplasmic Njun.

Given that junctin appears to regulate RyRs through its N- and C-terminal domains, we examined the combined effect of cytoplasmic Njun plus luminal Cjun. Njun added first to the cytoplasmic solution increased RyR activity (1st black bin, Fig. 5A–F) to levels significantly greater than luminal FLjun (cyan bins). Subsequent luminal addition of Cjun reduced the open probability (2nd black bin), to levels that remained greater than control, but were not significantly different from those with luminal FLjun.

In the complementary experiment, *P*_o_ fell when luminal Cjun was added first, but then increased with cytoplasmic Njun addition, although the activity remained less than that for the control (grey bins, Fig. 5A–F). This was in contrast to the greater than control activity with cytoplasmic Njun alone (Fig. 5, black bins, and Fig. 3) or with luminal FLjun (Fig. 5, cyan bins). Similar changes in open probability with FLjun (luminal), or Njun (cytoplasmic) followed by Cjun (luminal), were mainly due to changes in closed times (Fig. 5C,F, red ovals). These results suggest that increased RyR activity with luminal FLjun might depend on the cytoplasmic N-tail binding first, followed by interactions with the luminal C-domain.

The data in Fig. 5 support our assumptions (1) that FLjun inserts into the bilayer, as do RyRs and variety of other membrane proteins, and (2) that after insertion, the short N-terminal domain is exposed to the cytoplasmic solution, while the highly charged C-terminus remains on the luminal side. The consistent increase in RyR activity after FLjun addition to the luminal solution suggests that FLjun routinely inserts into the bilayer in the same orientation.

### Regions in RyR1 that interact with junctin

**Fig. 6.**
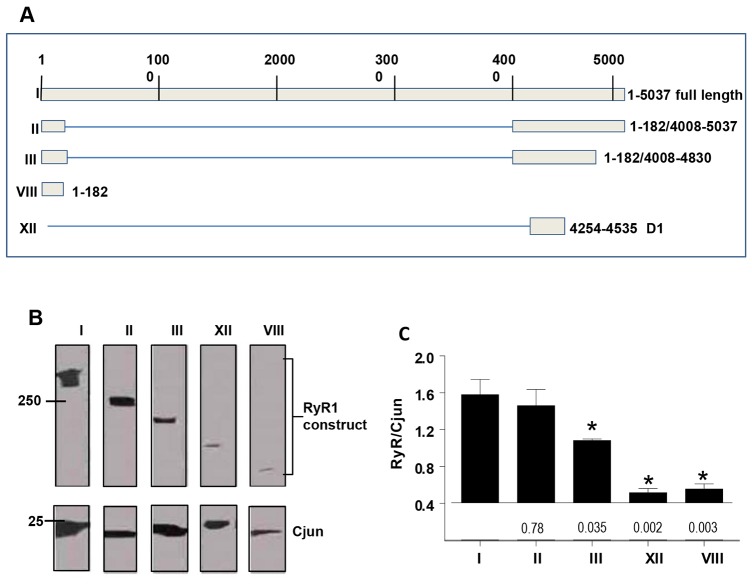
**Regions of RyR1 that interact with Cjun.** (A) Fragments of RyR1 examined for Cjun binding. Boxes indicate recombinant fragments. (B) Co-immunoprecipitation of recombinant RyR1 and RyR1 fragments by Cjun bound to the anti-junctin antibody conjugated to protein-A/G–agarose. RyR fragments were identified by using anti-GFP antibody, Cjun was identified by using anti-junctin antibody. (C) Mean±s.e.m. density of RyR1 constructs, relative to the density of Cjun in the same lane as shown in B (*n* = 3–5). *P<0.05 for differences between WT RyR1 and indicated fragment, *P*-values are given beneath the bins.

**Fig. 7.**
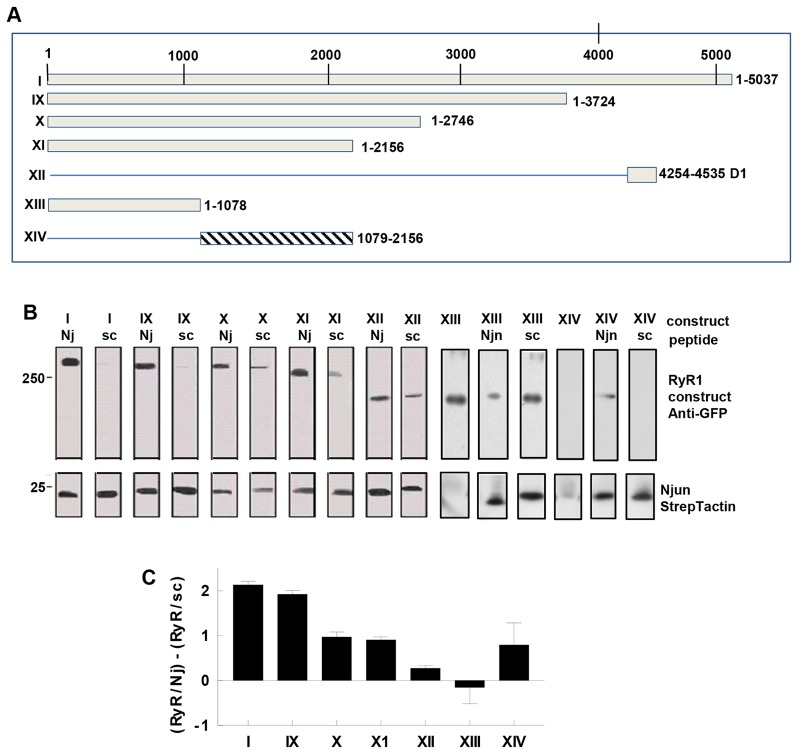
**Regions of RyR1 that interact with Njun.** (A) Fragments of RyR1 (boxes) examined for Njun binding. (B) Affinity chromatography of RyR1 or RyR1 fragments bound to biotinylated Njun (Nj) or scrambled Njun (sc) peptide associated with streptavidin–agarose. RyR fragments identified by anti-GFP antibody, biotinylated peptides detected by StrepTactin–HRP conjungate. The RyR constructs are listed in the upper row above each lane. Three lanes are shown for RyR1 XIII and XIV, the first the result of adding XIII and XIV to streptavidin agarose in the absence biotinylated peptide, showing non-specific binding of XIII, but not XIV to beads. (C) Mean±s.e.m. density of each RyR1 band relative to the density of Njun (RyR1:Njun) minus RyR1 density relative to scrambled Njun (RyR1:scrambled Njun) in each lane (*n* = 3 for each), to remove the fraction of binding that might be non-specific as indicated by scrambled Njun binding.

Cjun- and Njun-binding sites on RyR1 were examined using deletion mutations of rabbit RyR1 (Fig. 6A; Fig. 7A, see Materials and Methods). Although RyR preparations were obtained from different species for reasons of availability and compatibility with affinity chromatography materials, the results appear to be largely species independent. It is notable that FLjun, Cjun and Njun bound to full-length RyR1 from rabbit skeletal muscle, full length recombinant mouse RyR2 (Fig. 1C–F), full-length recombinant rabbit RyR1 (Fig. 6B,C; Fig. 7B,D) and interacted consistently with full-length RyR2 from sheep heart (Figs 2–5).

#### RyR1-binding sites for Cjun

Cjun bound to RyR1 constructs II and III, containing RyR1 N-terminal residues 1–182 plus C-terminal residues 4008–4830, but did not bind substantially to cytoplasmic residues 1–182 alone (VIII) or to residues 4254–4535 (XII) containing divergent region I (D1) (Fig. 6B,C). Cjun binding to construct III was significantly less than binding to I or II, indicating that residues 4831–5837, which contain the pore loop, influence Cjun binding to RyR1. However the major RyR1-binding sites for Cjun appear to be located between residues 4535–4830, containing the S1–S2 linker (as defined by Ramachandran et al., 2013) and previously known as the M5–M6 linker (Zissimopoulos and Lai, 2007; Zorzato et al., 1990), a region in human RyR2 that binds to junctin (Altschafl et al., 2011).

#### RyR1-binding sites for Njun

Njun bound to all RyR1 constructs containing residues 182–2156 (I, IX, X and XI; Fig. 7B,C), with little binding to the scrambled Njun peptide. Construct XII bound similarly to Njun and the scrambled Njun peptide (Fig. 7B), suggesting non-specific binding. None of the I, IX, X, XI or XII constructs bound to neutravidin–agarose in the absence of peptide. However the 1–1078 construct (XIII) bound to neutravidin agarose and to the scrambled Njun peptide in greater amounts than Njun (Fig. 7B). Construct 1079–2156 (XIV) bound to Njun only, not to the scrambled Njun peptide or neutravidin agarose (Fig. 7B), indicating highly specific binding. To asses specific Njun binding, the scrambled Njun peptide densities for each construct were subtracted from Njun densities (Fig. 7C). The results indicate that an N-terminal RyR1-binding site for Njun lies between residues 1097 and 2156 (XIV). Although Njun appears not to bind specifically to residues RyR1 1–1097 (XIII), a conclusion for this construct is complicated by its binding to streptavidin agarose.

### RyR-binding residues in Cjun

**Fig. 8.**
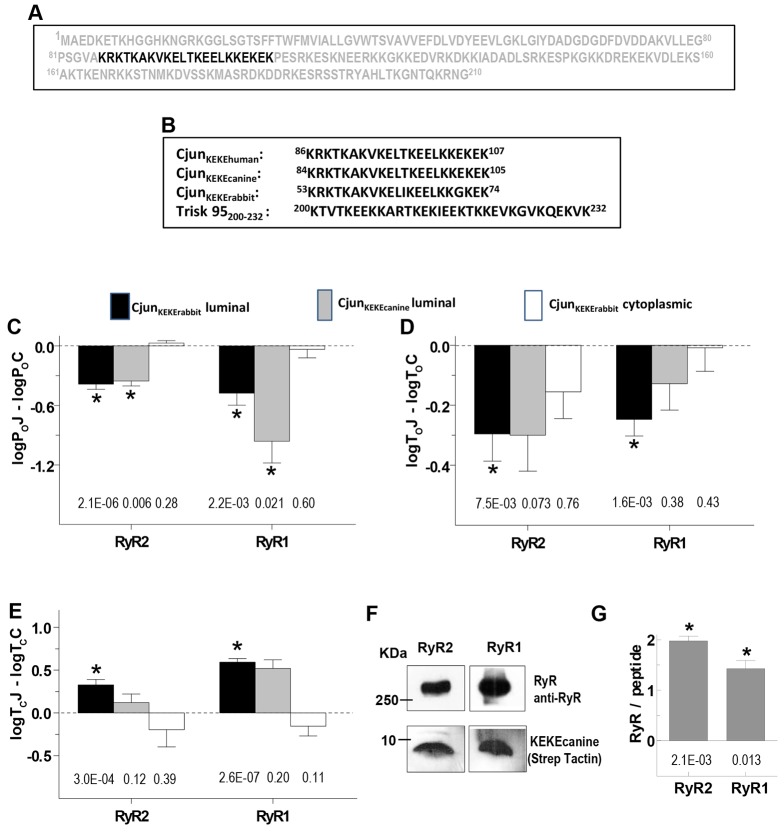
**A region of junctin that interacts with RyRs.** (A) Sequence of human junctin with KEKE motif residues (86–107) highlighted. (B) Conservation of the KEKE motif sequence in human, canine and rabbit junctin and comparison with the active KEKE motif in skeletal triadin. Rabbit junctin is not fully sequenced, so residue numbers are for the partial sequence (accession number AAF37204). (C–E) Effects of the KEKE peptides on average log*P*_o_J–log*P*_o_C, log*T*_o_J–log*T*_o_C, and log*T*_C_J–log*T*_C_C (as defined in Fig. 2) for: rabbit KEKE peptide on the luminal side of RyR2 (*n* = 16) and RyR1 (*n* = 12) (black bins); canine KEKE peptide [included because Cjun was derived from canine junctin (see Materials and Methods)] on the luminal side of RyR2 (*n* = 4) and RyR1 (*n* = 4) (grey bins); and rabbit KEKE peptide on the cytoplasmic side of RyR1 (*n* = 8) and RyR2 (*n* = 8) (white bins). *P<0.05 between control parameters and parameter with KEKE constructs; *P*-values are given below bins for each construct. (F) Affinity chromatography showing RyR2 and RyR1 binding to canine KEKE-peptide–streptavidin–agarose. (G) Mean±s.e.m. densitometry data showing consistent binding of RyR1 (*n* = 3) and RyR2 (*n* = 3) to canine KEKE-peptide–streptavidin–agarose. *P<0.05 for ratios significantly greater than zero; *P*-values are given under bins.

Cjun binds to the pore loop of RyR2 (Altschafl et al., 2011), a region of high charge density that, in RyR1, also binds to triadin (Goonasekera et al., 2007). A KEKE motif in triadin has been implicated in binding to RyR1 (Wium et al., 2012); hence, we explored RyR interactions with a similar KEKE motif that is present in the C-terminal junctin residues 86–107 (Fig. 8A,B). The KEKE peptides (Fig. 8B), like Cjun, added to the luminal solution, inhibited RyR channels with significant increases in closed times and decreases in open times (Fig. 8C–E). This contrasts with RyR1 activation produced by the triadin KEKE_200–232_ peptide (Wium et al., 2012), which has a similar KEKE content (Fig. 8B). These opposite actions were not explored further, but might be due to the slightly different KEKE sequence in triadin and junctin, or to the extended hydrophobic C-terminal tail of the triadin peptide allowing additional RyR interactions. In contrast to Cjun, and similar to the triadin KEKE_200–232_ peptide (Wium et al., 2012), the KEKE peptide in the cytoplasmic solution had no effect on RyR channels (Fig. 8C–E). In conclusion, the KEKE motif in the junctin 86–107 region can bind to RyR1 and RyR2 and reproduce the effects of Cjun on the luminal side of the channels. Affinity chromatography reveals a strong association between the KEKE peptide and purified RyR1 or RyR2 (Fig. 8F,G).

## DISCUSSION

Here, we report a new *in vitro* interaction between the N-terminal cytoplasmic residues of junctin and the cytoplasmic domains of RyR1 and RyR2, in addition to the expected luminal interactions. We show that a binding site for the N-terminal domain of junctin resides in residues 1078–2156 of RyR1 and suggest that this cytoplasmic binding must be established before the luminal interaction in order to replicate the normal regulation of RyR channels by junctin. In addition to the cytoplasmic binding site, the results indicate that there is a luminal RyR1 binding site for junctin between residues 4535–4830, which is the region containing the S1–S2 linker, which was already known to bind to junctin in human RyR2 (Altschafl et al., 2011). The N- and C-terminal parts of this linker region are homologous in rabbit RyR1 and RyR2, although the overall similarity in residues 4535–4830 is only 75% (BLASTP 2.2.30+; supplementary material Table S4). The very similar effects of junctin on RyR1 and RyR2 activity indicate that the functional binding is to regions conserved in the two RyR isoforms. Finally, this work reveals differences between the functional outcome of junctin and triadin interactions with RyRs, supporting the idea that two ‘anchoring’ proteins have independent roles in Ca^2+^ homeostasis. These differences include (1) several binding sites for junctin, in contrast to a single triadin-binding site in the RyR pore loop (Goonasekera et al., 2007), (2) RyR activation by the N-terminal domain of junctin, in contrast to inhibition by N-terminal triadin residues (Groh et al., 1999), and (3) the luminal KEKE-rich region of junctin (amino acids 86–107) inhibits RyRs, whereas the equivalent triadin region (amino acids 200–232) activates RyR1 (Wium et al., 2012). These differences are consistent with reports that junctin communicates signals from CSQ1 (CASQ1) to RyR1 (Wei et al., 2009a), whereas triadin influences excitation–contraction coupling (Goonasekera et al., 2007).

### The significance of N-terminal junctin binding to RyRs

The results suggest that the cytoplasmic site is physiological as it appears to be occupied by the endogenous junctin N-tail in the native channels. As noted above, we suggest that when FLjun is added to the luminal bilayer solution, its hydrophobic transmembrane domain (residues 23–43) inserts into the bilayer such that the short N-terminus is located in the cytoplasmic solution and the C-terminal domain remains in the luminal solution (Wei et al., 2009a). Given that the actions of luminal FLjun can only be replicated when Njun is added first to the cytoplasmic solution and then Cjun to the luminal solution, we suggests that FLjun insertion into the bilayer might allow the cytoplasmic interaction to anchor FLjun to the RyRs and facilitate the luminal interaction.

### Similar actions of junctin on RyR1 and RyR2

It is notable that FLjun, Njun and Cjun bind similarly to cardiac and skeletal RyRs, with little RyR isoform dependence in the functional effect of either cytoplasmic or luminal interactions. This is not surprising as the same junctin splice variant of the aspartate-β-hydroxylase gene is expressed in the heart and skeletal muscle. Although there is considerable overall sequence diversity between RyR1 and RyR2, some regions of high similarity exist. One explanation for the similar results is that the junctin-binding sites are in regions of sequence similarity in RyR1 and RyR2. Curiously, CSQ1 inhibits RyR1 by binding to junctin (Wei et al., 2009a), whereas CSQ2 (CASQ2) activates RyR1 and RyR2 (Wei et al., 2009b). If RyR1 or RyR2 activation by CSQ2 is also mediated through junctin, CSQ1 and CSQ2 must cause different conformational changes in junctin.

### Multiple junctin-binding sites on RyRs

The C-terminal domain of junctin binds to the S1–S2 (M5–M6) linker and the pore loop of RyR2 (Altschafl et al., 2011) and is likely to bind to the same regions in RyR1. We found that the residues including the largely uncharged S1–S2 linker region of RyR1 bound to Cjun. The KEKE motif could well interact with charged residues in the pore loop as does the similar KEKE motif in triadin (Goonasekera et al., 2007; Wium et al., 2012). Both the cytoplasmic RyR binding site and S1–S2 linker binding would help sustain full-length junctin binding to mutant RyRs with acidic residues in the pore loop replaced by alanine (Goonasekera et al., 2007). KEKE motifs in junctin and triadin are implicated in binding not only to RyRs but also to CSQ. The same KEKE motif in triadin reportedly binds to both CSQ and the RyR, raising the possibility that both cannot bind to triadin at the same time (Dulhunty et al., 2009). That triadin cannot convey signals from CSQ1 to RyR1 could be explained if CSQ1 binding to triadin disrupted the association of triadin with RyR1 (Wei et al., 2009a). In contrast to triadin, junctin could convey signals from CSQ1 to RyR1 (Wei et al., 2009a), due to multiple RyR–junctin (this manuscript) and junctin–CSQ (Kobayashi et al., 2000) binding sites. Thus, junctin could remain anchored to RyR1, while also binding CSQ1 and allowing CSQ1 to influence RyR1 activity (Wei et al., 2009a).

There is a complex interaction between triadin, junctin, CSQ and RyRs in living cells. Our previous studies in myotubes (Goonasekera et al., 2007) and other studies in C2C12 cells (Wang et al., 2009) suggest that triadin is more involved in excitation–contraction coupling, whereas junctin supports resting RyR activity and CSQ regulation of RyRs (Wei et al., 2009a). However junctin knockout, triadin knockout or triadin and junctin double-knockout suggest that the triadin–CSQ complex has a greater impact on junctional structure than the junctin–CSQ complex (Boncompagni et al., 2012). It is likely that dynamic functional effects and long-term structural effects reflect different aspects of Ca^2+^ homeostasis and that the transgenic results are complicated by changes in structure and in expression of CSQ and other junctional proteins.

### The physiological role of junctin

It remains our hypothesis that junctin is involved in maintaining RyR activity at rest and in transmitting signals from CSQ to RyRs. This is a significant role given the deleterious effects of changes in resting RyR activity or ‘leak’ in causing myopathies such as central core disease and malignant hyperthermia in skeletal muscle (Dainese et al., 2009; Paolini et al., 2007) and in precipitating cardiac arrhythmia (Jiang et al., 2004; Terentyev et al., 2006). Indeed junctin-knockout and knockout or mutation of CSQ both increase channel leak and can lead to arrhythmia (Altschafl et al., 2011; Faggioni and Knollmann, 2012). In bilayer studies, experimental removal of CSQ2 from RyR2 increases the sensitivity of RyR2 to changes in luminal Ca^2+^, in a manner that could lead to arrhythmia (Dulhunty et al., 2012). Similar changes in sensitivity to luminal [Ca^2+^] are seen with junctin knockout (Altschafl et al., 2011) and are likely due to removal of the link to CSQ2.

### Conclusions

The results reveal a strong interaction between the N-terminal tail of junctin and the cytoplasmic side of RyR1 and RyR2 channels and indicate that this interaction dictates the functional consequences of the full-length protein binding to skeletal and cardiac RyR channels. A modulatory effect of the luminal C-terminal domain on the cytoplasmic N-terminal activation is essential to achieve the normal regulatory action of the full protein on channel activity. The multiple junctin-binding sites on RyR1 and RyR2 raise the unexpected possibility that the influence of this anchoring protein on RyR activity is modified by factors in the cytoplasm as well as in the SR lumen.

## MATERIALS AND METHODS

### Materials

DNA restriction and modifying enzymes were from New England Biolabs and Roche Applied Science; T4 DNA ligase was from Promega; anti-GFP from Roche; anti-RyR (34C, which recognizes both RyR1 and RyR2, DSHB) from the Developmental Studies Hybridoma Bank). The antibody against a junctin peptide (SKHTHSAKGNNQKRKN, with a cysteine added to the N-terminus and conjugated to KLH, GL Biochem, Shanghai) was from IMVS Veterinary Services, Australia. PCR primers were from GeneWorks, Australia.

### Species

RyR1, RyR2 and junctin was sourced from several different species for various reasons including amounts required, compatibility with affinity chromatography materials and availability. Identity and homology between isoforms and species where known are given in supplementary material Tables S1–S4.

### FLjun plasmids, expression and purification

Full-length canine junctin (amino acids 1–210) in the pBlueScript II SK vector (Jones et al., 1995) was sub-cloned into a pET15b.ep (mod) vector using *Nde*I/*/Bam*HI sites and the primers, forward, 5′-GGGGGGCATATGGCTGAAGAGACAAAG-3′ and reverse, 5′-CGGGATCCTCAGTTCTTCTTCTTC-3′.

The FLjun-pET15b.ep (mod) plasmid was transformed into *E. coli* BL21(DE3). Expression of the N-terminal His-tagged FLjun was induced by 0.6 mM isopropyl-β-D-thiogalactoside (IPTG) with 3–4 h incubation at 37°C. The cell pellet was resuspended in cold PBS (137 mM NaCl, 7 mM Na_2_HPO_4_, 2.56 mM NaH_2_PO4, pH 7.4) plus 1% Triton X-100, 5% glycerol and protease inhibitor cocktail tablet (Roche Diagnostics, Germany). The cells were lysed in 0.5 mg/ml lysozyme for 20 min on ice, sonicated and centrifuged twice at 15,000 ***g*** for 25 min, then the supernatant centrifuged at 150,000 ***g*** for 45 min. The final supernatant containing solubilised FLjun was incubated with Ni^2+^-nitrilotriacetic acid (NTA)–agarose plus 25 mM imidazole at 4°C for 2 h, the FLjun-poly-His-Ni^2+^-NTA–agarose complex washed in cold Buffer A (50 mM NaH_2_PO_4_, 300 mM NaCl, pH 8.0 with 1% Triton X-100, 5% glycerol and 25–35 mM imidazole) and the fusion protein eluted with Ni elution buffer (50 mM NaH_2_PO_4_, 300 mM NaCl, 250 mM imidazole, pH 8.0 with 1% Triton X-100 and 5% glycerol). The protein was concentrated and dialysed against MOPS buffer (20 mM MOPS, pH 7.4, 150 mM NaCl plus 0.2% Triton X-100 and 5% glycerol). Triton X-100 was removed by incubation with Bio-Beads SM-2 adsorbents (Bio-Rad) at room temperature for 1.75 h and the centrifugation (∼1000 ***g*** for 3 min). The supernatant containing purified FLjun was concentrated (centrifugal filter, Millipore) and stored at −80°C.

### Cjun plasmids, expression and purification

Cjun DNA (amino acids 46–210) was amplified from full-length canine junctin in the pBlueScript II SK vector (Jones et al., 1995), cloned into pGADT7 vector and sub-cloned from pGADT7-CJun into the pET15b.ep vector using *Nde*I/*Bam*HI sites using the primers, forward, 5′-CGGAATTCCTTGTTGATTATGAAGAAGTT-3′ and reverse, 5′-CGGGATCCTCAGTTCTTCCTCTCTGGT-3′. Cjun was also expressed in pHUE (Baker et al., 2005; Catanzariti et al., 2004) for removal of the His atge, and obtained from Cjun- pET15b.ep using the primers, forward, 5′- GACCCGCGGTGGACTTGTTGATTATGAAG-3′ and reverse, 5′- CGAAGCTT TCAGTTCTTCCTCTTC-3′.

The Cjun-pET15b.ep plasmid was transformed into *E. coli* BL21/DE3. Expression of the N-terminal His-tagged protein was induced by 0.6 mM IPTG. Cjun was purified from the soluble lysate fraction using Buffer A (50 mM phosphate buffer PH 8 and 300 mM NaCl) with Ni-agarose affinity chromatography. The solution was exchanged for 20 mM MOPS, pH 7.4 and 150 mM NaCl, then Cjun concentrated (centrifugal filter, Millipore) and stored at −80°C. Cjun-pHUE was also transformed and poly His-ubiquitin-tagged Cjun expressed in *E. coli* BL21DE3 (as Cjun-pET15b.ep). The poly His-ubiquitin tag was removed as described previously (Baker et al., 2005; Catanzariti et al., 2004) and Cjun purified, concentrated and stored at −80°C.

### RyR1 plasmids, expression and isolation

Numerals in parentheses refer to constructs defined in Fig. 6A and Fig. 7A. GFL (I) (RyR1 5′ EGFP tagged), Gbhat (II) (amino acids 1–182 and 4008-End) and SM1 (III) (amino acids 1–182 and 4008–4830) are as described previously (Bhat et al., 1997; Treves et al., 2002; Zorzato et al., 1990). SM2 (VIII) (amino acids 1–182): *Sal*I546/*Sal*I_polylinker_ removed from GFL and the remaining GFL re-ligated. (IX), amino acids 1–3724; (X), amino acids 1–2746; (XI), amino acids 1–2156; (XII), RyR1 amino acids 4254–4535; (XIII), amino acids 1–1078; and (XIV), amino acids 1079–2156 plasmids in pJ204, from DNA2.0 (Menlo Park, CA), were digested by *Hin*dIII and *Kpn*I and cloned in frame into the *Hin*dIII and *Kpn*I sites in pEGFP-C3 vector polylinker. RyR1 constructs were expressed in HEK293 cells and protein isolated (Goonasekera et al., 2007). Microsomal vesicles were prepared and stored at −80°C. Mouse RyR2, stably expressed in an inducible HEK293 cell line (Koop et al., 2008), was isolated with the same method as RyR1.

All FLjun, Cjun and RyR constructs were sequenced by the JCSMR Biomolecular Resource Facility.

### Peptide synthesis

The following peptides were synthesised by the JCSMR BRF, with purification using HPLC and mass spectroscopy: Njun, ^1^MAEETKHGGHKNGRKGGLSQSS^22^; scrambled Njun, STGENKGHGLSGKHKSEGRAMG; Cjun KEKE motif (canine), ^84^KRKTKAKVKELTKEELKKEKEK^105^; Cjun KEKE motif (rabbit) ^53^KRKTKAKVKELIKEELKKGKEK^74^.

Peptides for affinity chromatography were constructed with an N-terminal biotin tag. Rabbit KEKE motif peptides were tested because RyR1 and some FLjun were isolated from rabbit muscle. The canine KEKE motif was also tested because recombinant canine FLjun was also used (see isolation of FLjun below) and Cjun was derived from canine cardiac junctin. Note that the KEKE sequence is identical in canine junctin (Fig. 8) and human junctin (as used by Altschafl et al., 2011).

### SR vesicle isolation, RyR purification

Rabbit skeletal SR and sheep ventricular SR vesicles were isolated and RyRs purified as described previously (Wei et al., 2009b). Sucrose gradient fractions containing RyRs (Western blot detection) were concentrated, 15 µl aliquots frozen and stored at −80°C.

### Co-immunoprecipitation

Co-immunoprecipitation was used to assess Cjun binding to native or recombinant RyRs and RyR1 constructs (Beard et al., 2008; Wei et al., 2006). 100 µg of purified RyRs or 3 µg of Cjun were diluted to 100 µl in immunoprecipitation buffer (0.025 M Tris-HCl, 0.15 M NaCl, 0.001 M EDTA, 1% NP-40, 5% glycerol, pH 7.4) and pre-cleared by rotation for 2 h at 4°C with 100 µl of washed control agarose resin. 10 µg of anti-junctin was diluted to 100 µl in coupling buffer (0.01 M sodium phosphate, 0.15 M NaCl, pH 7.2), rotated with 25 µl of washed and equilibrated protein-A/G–agarose for 2 h at room temperature with addition of 3 µl sodium cyanoborohydride, then washed, quenched, and re-equilibrated with immunoprecipitation buffer. The antibody–agarose was then incubated overnight at 4°C with either 3 µg of the pre-cleared Cjun or with immunoprecipitation buffer alone, then incubated with 100 µg of pre-cleared RyR or RyR1 constructs for 2 h at room temperature. Each incubation was followed by three or four washes and centrifugation steps. In the reverse assay, 10 µg of 34C anti-RyR1 and RyR2 or -GFP antibody was diluted to 100 µl in coupling buffer, bound to protein-A/G–agarose, coupled with 10 µg of pre-cleared RyRs or RyR1 constructs, then incubated with 10 µg of pre-cleared Cjun. Proteins were eluted by boiling for 5 min in 15–20 µl of Laemmli sample buffer and 5 to 10 µl was loaded onto SDS-PAGE gels. Importantly, a constant volume was added for each series of experiments. Proteins were separated by SDS-PAGE, transferred onto polyvinyl difluoride (PVDF) membrane and immunoprobed with 34C anti-RyR1 and RyR2 or -GFP antibody to detect RyR, or with anti-junctin antibody to detect Cjun.

### NeutrAvidin agarose affinity chromatography

Binding of Njun and scrambled Njun to native or recombinant RyRs or RyR1 fragments was detected by affinity chromatography (Rebbeck et al., 2011; Wium et al., 2012). 100 µl of NeutrAvidin–agarose (Thermoscientific, Rockford, IL) was washed twice in wash buffer (PBS plus 0.1% SDS) and then resuspended to 100 µl in the same buffer. 100 µg of biotinylated peptide was diluted to 1 µg/µl in binding/wash buffer (PBS plus 0.1% SDS), added to 100 µl of agarose slurry, incubated for 2.5 h at room temperature, and then washed five times with centrifugation (1000 ***g*** for 3 min) in wash buffer. Purified RyR protein (90 µg) was diluted in wash buffer plus protease inhibitor (AEBSF, Sigma-Aldrich, USA, or Complete EDTA-free protease Inhibitor Cocktail, Roche Diagnostics, Germany), precleared with control agarose resin (200 µl) for 2 h at room temperature, incubated with NeutrAvidin–agarose-bound biotinylated peptide or NeutrAvidin–agarose alone overnight at 4°C, then washed five times as above. Protein was eluted at 65°C for 10 min in 15–20 µl of Laemmli sample buffer and 5 to 10 µl was loaded onto SDS-PAGE gels. Importantly, a constant volume was added for each series of experiments. Proteins were separated on SDS-PAGE, transferred onto PVDF membrane and immunoprobed with 34C anti-RyR1 and -RyR2 or -GFP antibody to detect RyR protein, or with StrepTactin to detect biotinylated peptides.

### Isolation of FLjun

Junctin was isolated from rabbit skeletal junctional face membrane (JFM) using SDS preparative gel electrophoresis in a Bio-Rad 491 prep cell (Wei et al., 2009a). JFM was solubilized in 1%/0.5% CHAPS/PC, 1 mM DTT, 1 M NaCl, 20 mM MOPS, and 200 µM EGTA pH 7.4 with protease inhibitors, on ice for 1 h, then centrifuged at 135,000 ***g*** for 15 min. The supernatant was diluted with sample buffer (188 mM Tris-HCl, pH 6.8, 30% glycerol, 6% SDS, 0.06% bromophenol blue, and 15% β-mercaptoethanol), and boiled for 5 min before loading onto a 6.5-cm vertical cylindrical polyacrylamide gel. The resolving gel contained 12% acylamide/bis (37.5∶1), 0.375 M Tris-HCl pH 8.8, 0.025% ammonium persulphate (APS), and 0.025% *N,N,N,N*-Tetramethylethylenedianime (TEMED). A stacking gel, double the sample volume, contained 4% acylamide/bis (37.5∶1), 0.125 M Tris-HCl pH 6.8, 0.05% (APS), and 0.1% TEMED. Electrophoresis was at 40 mA in 25 mM Tris-HCl, 191 mM glycine, 0.1% SDS, pH 8.3. A total of 80 2-ml fractions were collected at a flow of 1 ml/min and analysed by SDS-PAGE and immunoblotting. Junctin was renatured after precipitation in 400 mM KCl for 20 min at room temperature with gentle agitation. The fractions were centrifuged (∼15,800 ***g***) for 15 min, the protein eluted and re-folded by detergent exchange in 0.2% Triton X-100, 20 mM MOPS, 150 mM KCl, 200 µM EGTA, pH 7.4 plus protease inhibitors, at room temperature for 1 h, then centrifuged (∼15,800 ***g***) for 10 min to remove SDS-KCl precipitate. The supernatant protein was concentrated and exchanged with 20 mM MOPS pH 7.4, 150 mM NaCl, 200 µM EGTA plus protease inhibitors) using Millipore Amicon Ultra-4 centrifugal filters. Purified junctin (0.2–0.4 mg/ml) was stored at −70°C.

Note that FLjun isolated from rabbit skeletal muscle was initially used in binding and bilayer experiments. However, the extraction was lengthy and yields were very low and we obtained better yields with recombinant canine junctin. There was no observable difference between results obtained with the recombinant FLjun and FLjun isolated from skeletal muscle and both was combined in average data.

### Single-channel recording and analysis

RyRs were incorporated into lipid bilayers and channel activity recorded using standard techniques (Beard et al., 2008; Wium et al., 2012). Cis (cytoplasmic) Ca^2+^ was adjusted to 1 µM or 100 nM with [BAPTA] determined using a Ca^2+^ electrode. Trans [Ca^2+^] was 1 mM throughout. Open probability (*P*_o_), mean open (*T*_o_), mean closed (*T*_c_), time or fractional mean current (*I*′_F_) were measured (Beard et al., 2008; Wei et al., 2006). Open and closed time constants, determined using logged bin analysis (Sigworth and Sine, 1987; Tae et al., 2011), were assigned to τ_O1_ or τ_C1_ (1–10 ms), τ_O2_ or τ_C2_ (10–50 ms) or to τ_O3_ or τ_C3_ (50–500 ms).

### Ca^2+^ release from SR vesicles

Extravesicular Ca^2+^ was measured spectrophotometrically at 710 nm, using the Ca^2+^ indicator antipyrylazo III was determined following Ca^2+^ loading and block of the Ca^2+^ pump with thapsigargin (Hanna et al., 2011; Jalilian et al., 2008).

### Statistics

Data are presented as mean±s.e.m. and significance was evaluated using paired or unpaired Student's *t*-test or ANOVA as appropriate. The number of observations (*n*) are given. As similar results were obtained at +40 mV and −40 mV in bilayer experiments, data for each potential was normally included in average data (i.e. two observations for each channel under each condition). To reduce effects of normal variability in the control parameters for RyRs, data with junctin constructs (*P***_o_**J, *T***_o_**J, *T***_c_**J) are expressed relative to control (*P***_o_**C, *T***_o_**C, *T***_c_**C) (e.g. log*P***_o_**J−log*P***_o_**C).

## Supplementary Material

Supplementary Material
