# Supplementary material for: A new cytoplasmic interaction between junctin and ryanodine receptor Ca2+ release channels
Source: J Cell Sci. 2015 Mar 1;128(5):951–63. doi: 10.1242/jcs.160689 (PMC4342579; doi:10.1242/jcs.160689)
Supplement: Supplementary Material [file supp_128_5_951__index.html]

A new cytoplasmic interaction between junctin and ryanodine receptor Ca2+ release channels — Supplementary Material 

# A new cytoplasmic interaction between junctin and ryanodine receptor Ca2+ release channels

## JCS160689 Supplementary Material

**Files in this Data Supplement:**

- **Supplementary Material**
