# Supplementary material for: A new cytoplasmic interaction between junctin and ryanodine receptor Ca2+ release channels
Source: J Cell Sci. 2015 Mar 1;128(5):951–63. doi: 10.1242/jcs.160689 (PMC4342579; doi:10.1242/jcs.160689)
Supplement: Supplementary Material [file supp_128.5.951_JCS160689.pdf]

Supplementary Table S1 – Cardiac junctin constructs – Note that sheep cardiac junctin is only partially sequenced.
Therefore comparison of identity and homology is given with the equivalent region of human and canine cardiac junctin in **A**.
Comparison of identity and homology of full human with canine junctin in **B**.
Comparison of canine Cjun identity and homology with human or rabbit Cjun in **C**.
Identity and homology values obtained using BLASTP 2.2.30+.

**A FLjun**	Corresponding canine FLjun			Corresponding human junctin	
	identity	homology		identity	homology
Partial rabbit cardiac FLjun	74%	83%		77%	84%

**B FLjun**	Canine junctin	
	identity	homology
Human junctin	85%	91%

**C Cjun**	Equivalent human Cjun			Equivalent rabbit Cjun	
	identity	homology		identity	homology
Canine Cjun (45-210)	83%	90%		72%	82%

Supplementary Table S2. Comparison of sequence identity and homology in indicated species.
RyR1 (**A**) or RyR2 (**B**)

A

RyR1	Human		Rabbit		Sheep		Canine		Mouse	
	identity	homology	identity	homology	identity	homology	identity	homology	identity	homology
Human	100%	100%	96%	97%	93%	94%	97%	97%	95%	97%
Rabbit	96%	97%	100%	100%	94%	95%	97%	97%	96%	97%
Sheep	93%	94%	94%	95%	100%	100%	94%	95%	92%	94%
Canine	97%	97%	97%	97%	94%	95%	100%	100%	97%	98%
Mouse	95%	97%	96%	97%	92%	94%	97%	98%	100%	100%

B

RyR2	Human		Rabbit		Sheep		Canine		Mouse	
	identity	homology	identity	homology	identity	homology	identity	homology	identity	homology
Human	100%	100%	99%	99%	98%	98%	98%	99%	97%	98%
Rabbit	99%	99%	100%	100%	97%	98%	98%	98%	97%	98%
Sheep	98%	98%	97%	98%	100%	100%	98%	99%	96%	98%
Canine	98%	99%	98%	98%	98%	99%	100%	100%	97%	98%
Mouse	97%	98%	97%	98%	96%	98%	97%	98%	100%	100%

Supplementary Table S3 – RyR1 constructs – regions in human and canine aligned with given rabbit residues

	Human			Canine	
	identity	homology		identity	homology
Rabbit 1-5037	96%	97%		97%	97%
Rabbit 1-182	98%	98%		98%	97%
Rabbit 1-2156	95%	97%		96%	97%
Rabbit 1-1078	97%	98%		97%	98%
Rabbit 1079-2156	93%	96%		94%	96%
Rabbit 4008-5037	92%	93%		95%	96%
Rabbit 4008-4830	91%	91%		93%	94%
Rabbit 4254-4535	92%	92%		89%	90%

Supplementary Table S4. Comparison of sequence identity and homology of Rabbit RyR1residues 4536-4830 with equivalent RyR2 region in listed species.

RyR2	Rabbit RyR1 4536-4830	
	identity	homology
Sheep	63%	76%
Dog	64%	76%
Human	63%	76%
Rabbit	63%	75%
Mouse	63%	75%
